# Isolation and Characterization of *AGAMOUS-Like* Genes Associated With Double-Flower Morphogenesis in *Kerria japonica* (Rosaceae)

**DOI:** 10.3389/fpls.2018.00959

**Published:** 2018-07-12

**Authors:** Jiang Ma, Xiangling Shen, Zhixiong Liu, Dechun Zhang, Wen Liu, Hongwei Liang, Yubing Wang, Zhengquan He, Faju Chen

**Affiliations:** ^1^Key Laboratory of Three Gorges Regional Plant Genetics & Germplasm Enhancement (CTGU)/Biotechnology Research Center, China Three Gorges University, Yichang, China; ^2^Forestry College, Beijing Forestry University, Beijing, China; ^3^College of Horticulture and Gardening, Yangtze University, Jingzhou, China

**Keywords:** double-flower, *AGAMOUS*, *Kerria japonica*, transposon, floral development

## Abstract

Double-flower phenotype is more popular and attractive in garden and ornamental plants. There is great interest in exploring the molecular mechanisms underlying the double-flower formation for further breeding and selection. *Kerria japonica*, a commercial ornamental shrub of the Rosaceae family, is considered an excellent system to determine the mechanisms of morphological alterations, because it naturally has a single-flower form and double-flower variant with homeotic conversion of stamens into petals and carpels into leaf-like carpels. In this study, *Sf-KjAG* (*AGAMOUS* homolog of single-flower *K. japonica*) and *Df-KjAG* (*AGAMOUS* homolog of double-flower *K. japonica*) were isolated and characterized as two *AGAMOUS* (*AG*) homologs that occur strictly in single- and double-flower *K. japonica*, respectively. Our sequence comparison showed that *Df-KjAG* is derived from ectopic splicing with the insertion of a 2411 bp transposon-like fragment, which might disrupt mRNA accumulation and protein function, into intron 1. Ectopic expression analysis in *Arabidopsis* revealed that *Sf-KjAG* is highly conserved in specifying carpel and stamen identities. However, *Df-KjAG* did not show any putative C-class function in floral development. Moreover, yeast-two-hybrid assays showed that Sf-KjAG can interact with KjAGL2, KjAGL9, and KjAP1, whereas Df-KjAG has lost interactions with these floral identity genes. In addition, loss-of-function of *Df-KjAG* affected not only its own expression, but also that of other putative floral organ identity genes such as *KjAGL2, KjAGL9, KjAP1, KjAP2, KjAP3*, and *KjPI*. In conclusion, our findings suggest that double-flower formation in *K. japonica* can be attributed to *Df-KjAG*, which appears to be a mutant produced by the insertion of a transposon-like fragment in the normal *AG* homolog (*Sf-KjAG*) of single-flower *K. japonica*.

**Highlights:** *Sf-KjAG* and *Df-KjAG* are different variations only distinguished by a transposon-like fragment insertion which lead to the evolutionary transformation from single-flower to double-flowers morphogenesis in *Kerria japonica*.

## Introduction

Transposon elements (TEs) refer to segments of DNA that can integrate at many different sites along a chromosome (Finnegan, 1985; Kidwell, 2002). In many plants, TEs comprise a major portion of the genome and increase genetic and functional diversity, which might provide more selective potential in plant evolution and domestication (Hirsch and Springer, 2017). In flowering plants, members of the MADS-box transcription factor gene family are known to regulate floral organ development, but few cases of genetic diversity and floral development caused by TE insertion in MADS-box family genes have been reported (Becker and Theissen, 2003; Bennetzen, 2005; El Baidouri et al., 2014).

In *Arabidopsis, AGAMOUS* (*AG*), encoding a MADS-box transcription factor, plays crucial functions in floral development, such as regulating stamen and carpel morphogenesis, limiting floral meristem determinacy, and antagonizing A-class genes (Coen and Meyerowitz, 1991; Soltis et al., 2002; Heijmans et al., 2012; Soltis and Soltis, 2014). Over the long term, *AG* homologous genes in different taxa have undergone sub-functionalization or neo-functionalization, but their functions in regulating the development of reproductive organs are broadly conserved, both in angiosperms and gymnosperms (Winter et al., 1999; Kyozuka and Shimamoto, 2002; Kramer et al., 2004; Zhang et al., 2004, 2015; Arrington, 2006). Mutation or aberrant expression of *AG* orthologous genes is often involved in double-flower formation (Bowman, 1997; Benedito et al., 2004; Dong et al., 2007; Hsu et al., 2010; Liu et al., 2013; Tanaka et al., 2013; Noor et al., 2014; Sun et al., 2014; Wang et al., 2015; Zhang et al., 2015). For example, loss-of-function of *AG* in *Arabidopsis* converts stamens into petals and replaces carpels with another *ag* mutant flower, known as double-flower (Bowman et al., 1991; Meyerowitz, 1994). In *Nigella damascena*, simultaneous downregulation of *NdAG1* and *NdAG2* results in the transformation of stamens into petals and carpels into sepals, as well as the loss of floral determinacy (Wang et al., 2015). Silencing of *TAG1* in tomato also causes double-flower formation (Dong et al., 2007). Simultaneous silencing of two C-class MADS-box genes, pMADS3 and FBP6, produces double-flower in *Petunia hybrid* (Noor et al., 2014). In *Camellia japonica*, the shift of *CjAG* expression boundary is associated with double-flower formation (Sun et al., 2014). In *Prunus lannesiana*, exon skipping of *PrseAG* results in the complete loss of AG motifs I and II in the C domain, which leads to double-flower formation (Liu et al., 2013). In *Magnolia stellata*, alternative splicing of *MastAG* generates three transcripts with divergent functions, which might result in the formation of multiple petals in *M. stellata* flowers (Zhang et al., 2015).

Although many double-flower phenotypes are noted in natural and domesticated species, the molecular mechanism underlying double-flower formation has only been investigated in few species. In addition, double-flower formation caused by TE insertion in *AG* orthologous genes was previously observed only in three ornamental plants, including Japanese morning glory (Nitasaka, 2003), *Thalictrum thalictroides* cultivar ‘Double White’ (Galimba et al., 2012) and *Gentiana scabra* (Nakatsuka et al., 2015). *Kerria japonica*, a common ornamental deciduous shrub in Rosaceae, has a single-flower form and a double-flower variant with homeotic conversion of stamens into petals and carpels into leaf-like carpels, and loss of floral meristem determinacy. Based on these observed phenotypes, we identified the formation of double-flower in *K. japonica* as a candidate for the loss of C-class function. In this study, we isolated two *AG*-like genes, *Sf-KjAG* from single-flower *K. japonica* and *Df-KjAG* from double-flower *K. japonica*. Detailed sequencing and expression analyses of *Sf-KjAG* and *Df-KjAG* were performed. Further, functional analyses of *Sf*-*KjAG* and *Df-KjAG* under the control of the cauliflower mosaic virus (CaMV) 35S promoter were conducted in the wild-type and homozygous *ag-1* mutant *Arabidopsis*. The results provided strong evidences of high functional conservation of the *AG* ortholog in *K. japonica* in specifying carpel and stamen identities and revealed the molecular mechanism of double-flower formation in *K. japonica*, as well as novel information regarding the involvement of a transposon in double-flower formation and evolution.

## Materials and Methods

### Plant Materials

Flower bud samples of single- or double-flower *K. japonica* were collected from Shengnongjia forest district and Beijing Forestry University. The samples were then frozen immediately in liquid nitrogen and stored at -80°C until use.

The *Arabidopsis ag-1* mutant line (CS3086) in an ecotype Landsberg background was obtained from the *Arabidopsis* Biological Resource Center, Ohio State University, Columbus, OH, United States.

### Rapid Amplification of cDNA Ends

Total RNA was extracted separately from floral buds of the single- and double-flower *K. japonica* by using the EASYspin plant RNA Extraction kit (Aidlab, China) following manufacturer instructions. The 3′-full rapid amplification of cDNA ends (RACE) Core Set Version 2.0 kit (TaKaRa, Japan) was used to synthesize the 3′RACE cDNA from 2 μg of DNase I-treated RNA by using 3′RACE adaptor primer and PrimeScript RTase. The 3′ portion of the *AG* homolog from single-flower *K. japonica* was isolated, which yielded a 1038 bp fragment that was amplified from single-flower *K. japonica* 3′RACE cDNA (forward primer, 3′AGGSPF1; reverse primer, 3′RACE outer primer). The 5′ RACE cDNA was synthesized from 1 μg of DNase I-treated RNA by using random primer and PrimeScript RTase by using the 5′-Full RACE Kit (TaKaRa, Japan). The 5′ portion of the fragment was then amplified using a pair of specific primers (5′AGGSPR1 and the 5′RACE outer primer). These PCR products were cloned into a pMD18-T vector and then sequenced. The cloning procedure of double-flower *K. japonica AG* homolog was similar to that of single-flower *K. japonica*. The accuracy and integrity of *AG* homologous cDNA sequences from *K. japonica* were validated by designing primers to amplify full-length CDS (primers Sf/Df-KjAGF and Sf-KjAGR for single-flower *K. japonica AG* homolog; primers Sf/Df-KjAGF and Df-KjAGR for double-flower *K. japonica AG* homolog). The products were cloned into pMD18-T vectors and then sequenced. The sequences were deposited in GenBank (accession numbers: KC476500 and KT884658).

### Cloning and Analysis of *Sf-KjAG* and *Df-KjAG* Genomic Sequence

Genomic DNA was extracted from young leaves of single- and double-flower *K. japonica* by using TaKaRa MiniBEST Plant Genomic DNA Extraction kit (Takara, Japan). The *Sf-KjAG* and *Df-KjAG* genomic DNA was amplified using Long PCR Enzyme Mix (Fermentas, Lithuania). Forward primer KjAG-GeDNA-F (in identical exon 1 of *Sf-KjAG* and *Df-KjAG*) and reverse primer KjAG-GeDNA-R (in exon 8 of *Sf-KjAG*) were used to amplify the full-length genomic DNA sequences of *Sf-KjAG* and *Df-KjAG*. The PCR products were cloned into pTOPO-TA vectors (Aidlab, China) and then sequenced. The sequences were deposited in GenBank (accession numbers: KT884659 and KT884660).

### Southern Blotting and Distribution Detection of *Sf-KjAG* and *Df-KjAG* Transcripts

Genomic DNA was extracted from the young leaves of single- and double-flower *K. japonica* using the TaKaRa MiniBEST Plant Genomic DNA Extraction kit (Takara, Japan) following the manufacturer’s protocol. Specific hybridization was confirmed by designing probe primers Kj-souF and Kj-souR in the lowest conserved C domain. PCR amplification was performed by separately using single- and double-flower *K. japonica* genomic DNA as template to detect primer specificity, and PCR products were cloned into a pTOPO-TA vector (Aidlab, China) and sequenced. The PCR products (727 bp consensus sequence in *Sf-KjAG* and *Df-KjAG* genomic DNA) amplified using Kj-souF and Kj-souR primers were labeled with digoxigenin by using DIG High Prime DNA Labeling and Detection Starter Kit II (Roche, Mannheim, Germany). Genomic DNA of single- and double-flower *K. japonica* was digested with SacI, HindIII, and EcoRI (TaKaRa, Japan); 15 μg of genomic DNA was loaded in each lane and separated on a 1% agarose gel, and transferred to HyBond-N+ nylon membrane (Amersham Biosciences, United Kingdom), and then fixed at 80°C for 0.5 h. Pre-hybridization, hybridization, stringency washes, and immunological detection were performed using a DIG High Prime DNA Labeling and Detection Starter Kit II (Roche, Mannheim, Germany).

In addition, multiplex PCR was performed to detect the expression distribution of *Sf-KjAG* and *Df-KjAG* transcripts in single- and double-flower *K. japonica*. Total RNA was extracted from 165 flower bud samples of single- and double-flower *K. japonica* collected from different sampling sites. The cDNA was synthesized from 1 μg of the DNase I-treated RNA by using oligo (dT)^18^ primer and MMLV Reverse Transcriptase. Multiplex PCRs were performed using primers Sf/Df-KjAG-F, Sf-KjAG-R, and Df-KjAG-R, and then detected on 1% agarose gels.

### Sequence Alignments and Phylogenetic Analysis

Deduced amino acid sequences for *Sf-KjAG* were subjected to BLAST analysis using sequence information from the GenBank database. During the BLAST searches, multiple C-class proteins from eudicot taxa were selected for alignment. During the construction of phylogenetic trees, we also introduced the A-, B-, D-, and E-class proteins. The GenBank accession numbers of the sequences used are listed in Supplementary Table S2. Full-length amino acid sequences comprising the MADS, I, K, and C domains of these genes were aligned using the ClustalW program under default settings. A phylogenetic tree was constructed using the Maximum likelihood method, including bootstrap analyses with 500 replicates, in MEGA6.0 software (Jones et al., 1992; Tamura et al., 2013).

### Semi-Quantitative Reverse Transcription-PCR and Real-Time PCR

For semi-quantitative reverse transcriptase (RT)-PCR analysis, total RNA was extracted separately from juvenile leaves, sepals, petals, stamens, and carpels of single-flower *K. japonica* and juvenile leaves, sepals, outermost petals, inner petals and leaf-like carpels of double-flower *K. japonica* at S9 and D9 stages by using an EASYspin plant RNA Extraction Kit (Aidlab, China). In double-flower *K. japonica*, the outermost 5 petals were considered as the outermost petals, and the remaining petals were considered as inner petals. After treatment with *DNase* I (TaKaRa, Japan), 1 μg of total RNA was used to synthesize the first-strand cDNA by using the oligo (dT)^18^ primer and MMLV Reverse Transcriptase. RT-PCR was performed using the following primers: RT-Sf-KjAGF and RT-Sf-KjAGR for *Sf-KjAG*, RT-Df-KjAGF and RT-Df-KjAGR for *Df-KjAG*, and RT-KjactinF and RT-KjactinR for *ACTIN* (reference control). Next, 1 μL of each cDNA sample was submitted to PCR as follows: 94°C for 5 min; thirty cycles of 30 s at 94°C, 30 s at 58°C, 30 s at 72°C; and a 10 min final extension at 72°C. The PCR products from all amplifications were analyzed using electrophoresis on a 1% agarose gel and photographed under ultraviolet light. Further, real-time quantitative PCR was performed to further confirm the expression abundance. The real-time quantitative PCR was performed using SYBR premix Ex Taq (Takara, Japan) and the following primers: qSf/Df-KjAG-F and qSf-KjAG-R for *Sf-KjAG*, qSf/Df-KjAG-F and qDf-KjAG-R for *Df-KjAG*, and qKjactin-F and qKjactin-R for *ACTIN* (reference control). The reaction conditions were as follows: 95°C for 3 min; forty cycles of 95°C for 15 s, and 60°C for 30 s. The abundance of other putative floral organ identity genes such as *KjAP1* (putative A-class), *KjAP2* (putative A-class), *KjAP3* (putative B-class), *KjPI* (putative B-class), *KjAGL2* (putative E-class), and *KjAGL9* (putative E-class) was determined by conducting real-time quantitative PCR expression analyses. The primers used for real-time quantitative PCR are listed in Supplementary Table S1.

Total RNA was extracted from bud samples of single- and double-flower *K. japonica* at different development stages. After treatment with *DNase* I (TaKaRa, Japan), 1 μg of total RNA was used to synthesize first-strand cDNA by using the oligo (dT)^18^ primer and MMLV Reverse Transcriptase. The real-time quantitative PCR of *Sf-KjAG* and *Df-KjAG* at different development stages was performed as the protocol mentioned before.

### Vector Construction and Transformation

Full-length coding sequences of *Sf-KjAG* and *Df-KjAG* were cloned into the binary vector pBI121 (BD Biosciences Clontech) by separately digesting with XbaI and SacI, and XbaI and SmaI, under the control of the CaMV 35S promoter in the sense orientation. The *35S::Sf-KjAG* and *35S::Df-KjAG* constructs were first transformed into *Agrobacterium tumefaciens* strain GV3101-90 and then into heterozygous *ag-1 Arabidopsis* (Landsberg *erecta*) by using the floral-dip method, as described by Clough and Bent (1998). In addition, pBI121 vector as a negative control was transformed into *Arabidopsis*.

The putatively transformed *Arabidopsis thaliana* seeds were germinated on solid 1/2 MS medium containing 35 μg/mL kanamycin. After the seeds were vernalized at 4°C for 2 days, they were transferred to a greenhouse under normal growth conditions (16 h light/8 h dark) at 22°C. After 2 weeks of screening, transformation-positive plants with green true leaves and long roots were transplanted into nutritional soil for cultivation. The transgenic plants were confirmed using quantitative real-time PCR (primers: qRT-Sf-KjAG-F and qRT-Sf-KjAG-R; qRT-Df-KjAG-F and qRT-Df-KjAG-R). The genotypes (wild-type, heterozygous *ag-1*, or homozygous *ag-1*) of transformed *Arabidopsis* were identified using the dCAPS methods (Neff et al., 1998). PCR was performed using a 5 min at 94°C denaturation step; thirty cycles of 94°C for 30 s, 52°C for 30 s, and 72°C for 30 s; and a final extension period of 5 min by using the primers ag-f and ag-r. Finally, 20 μL of the total PCR product (25 μL) in each reaction was digested using Afl II (TaKaRa, Japan) for 3 h and then separated on a 4% agarose gel, stained, and visualized under ultraviolet light.

### Yeast Two Hybrid Assay

Further evidence for *Df-KjAG* loss-of-function was provided by conducting yeast two hybrid (Y2H) assays by using Sf-KjAG, Df-KjAG, KjAGL2, KjAGL9, KjAP1, KjAP3, and KjPI, with *HIS3, AUR1-C, LacZ*, and *ADE2* serving as reporter genes by using the Matchmaker Gold Yeast Two-Hybrid System (Clontech, United States). If the co-transformed yeast grew and turned blue on the selective medium (lacking histidine and adenine and supplemented with aureobasidin A and X-a-Gal), the two tested genes were considered to interact with each other or form dimers. Full-length coding sequences of *Sf-KjAG, Df-KjAG, KjAGL2, KjAGL9, KjAP1, KjAP3*, and *KjPI* were fused into the pGADT7 vector with the GAL4 activation domain (AD), and the full-length coding sequences of *Sf-KjAG* and *Df-KjAG* were fused into the pGBKT7 vector with the GAL4-binding domain (BD). In addition, Sf-KjAGΔ (MADS domain of *Sf-KjAG* was deleted) was also fused into the pGBKT7 vector. All constructs were confirmed by sequencing and co-transformed into Y2HGold yeast competent cells according to the AD/BD combinations in **Figure 7Aii**. All the resultant combined AD/BD transformants were streaked onto synthetic dropout selective medium (SD/-Leu/-Trp medium or SD/-Leu/-Trp/-His/-Ade/+AbA/+X-a-Gal medium) for further observation. Parallel experiments were performed using pGBKT7-53 and pGADT7-T as positive controls and pGBKT7-Lam and pGADT7-T as negative controls.

## Results

### Morphological Comparison

**FIGURE 1:**
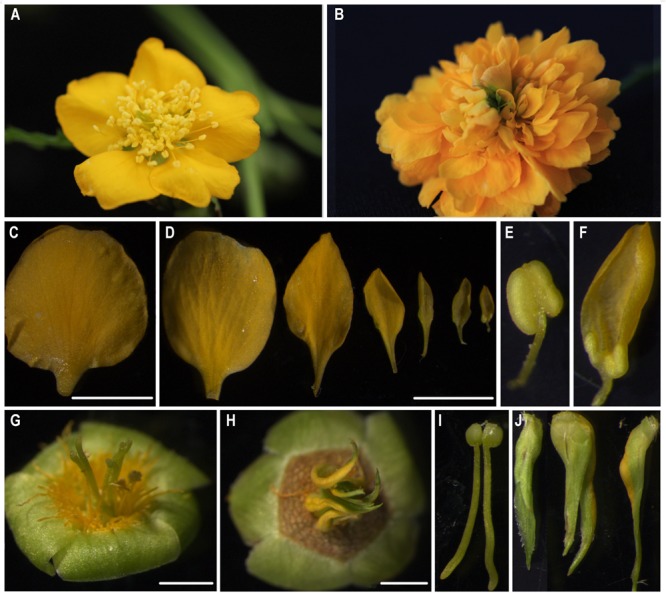
The morphological observation of single- and double-flower *Kerria japonica.* **(A)** The flower of single-flower *K. japonica.* **(B)** The flower of double-flower *K. japonica.* **(C)** The petals of single-flower *K. japonica.* **(D)** The petals from outerside to flower center of double-flower *K. japonica.* **(E)** The stamens of single-flower *K. japonica.* **(F)** The inner petals which remained the anther-like and filament-like structures in double-flower *K. japonica.* **(G,I)** The carpels of single-flower *K. japonica.* **(H,J)** The leaf-like carpels of double-flower *K. japonica.* Bars = 5 mm.

Single-flower *K. japonica* has four whorls of normal floral organs, which consist of five sepals, five petals, numerous stamens, and five carpels (**Figure 1A**). Conversely, in double-flower *K. japonica*, whorl 3 is replaced with numerous petals (**Figure 1B**), and whorl 4 is replaced with 6 to 10 leaf-like carpels (**Figures 1G–J**). Additional phenotypes can be observed within these two whorls: the petals of double-flowers dwindle down centripetally (**Figure 1D**); some petals in the center still retain filament-like and anther-like structures (**Figures 1D–F**); and partial leaf-like carpels are observed to be fused with the petals (**Figures 1B,H,J, 7B**).

### *Df-KjAG* of Double-Flower *K. japonica* Is a Truncated Mutant of *Sf-KjAG* of Single-Flower *K. japonica*

**FIGURE 2:**
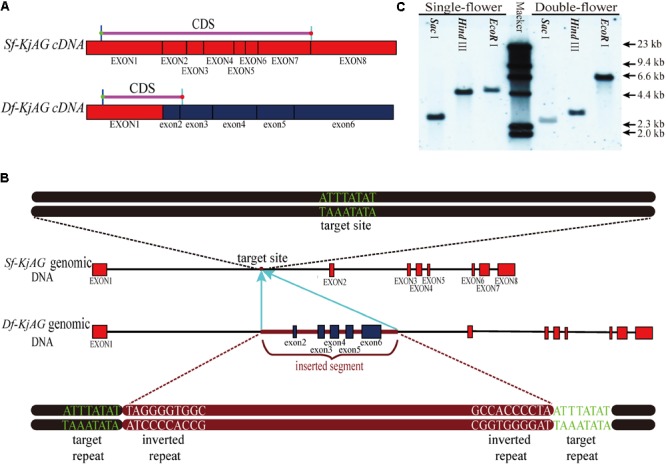
Sequence comparison of *Sf-KjAG* and *Df-KjAG*. **(A)** The diagram of *Sf-KjAG* and *Df-KjAG* cDNA sequences. **(B)** The diagram of *Sf-KjAG* and *Df-KjAG* genomic DNA sequences. Black lines represent introns, pink lines represent CDS, brown lines represent the inserted segment, red boxes represent normal exons, and blue boxes represent the exons from 2 to 6 of *Df-KjAG*, which are derived from the inserted segment. **(C)** Gene copy analysis by Southern blotting. Southern blotting analysis showed both *Sf-KjAG* in single-flower *K. japonica* and *Df-KjAG* in double-flower *K. japonica* are single copy.

Based on morphological observation and previous study findings, we speculated that the double-flower formation of *K. japonica* might be caused by the loss-of-function of C-class genes. Therefore, we separately isolated *AG* homologous genes from single- and double-flower *K. japonica*. Phylogenetic analyses revealed that the *AG* homolog cloned from single-flower *K. japonica* is nested in the euAG clade (Supplementary Figure S3). Therefore, the *AG* homolog isolated from single-flower *K. japonica* is referred to as *Sf-KjAG* (*AGAMOUS* homolog of single-flower *K. japonica*), and that isolated from double-flower *K. japonica* is referred to as *Df-KjAG* (*AGAMOUS* homolog of double-flower *K. japonica*). The cDNA alignment revealed that exon 1 of *Df-KjAG* was identical to that from *Sf-KjAG*, but lacked the I, K, and C domains. The *Sf-KjAG* transcript contains a 741 bp open reading frame encoding 246 amino acids (aa), which contain a 57 aa MADS domain, a 32 aa I domain, an 82 aa K domain, and a 57 aa C-terminal domain (Supplementary Figure S4). An 18 aa N-terminal extension, which is usually observed in euAG clade members (Kramer et al., 2004), was also found in *Sf-KjAG* (Supplementary Figure S4). Moreover, the Sf-KjAG protein contains two specific and highly conserved motifs of AG homologous proteins, AG motifs I and II. In contrast, the *Df-KjAG* transcript only contains a 303 bp open reading frame encoding a 100 aa protein, which only contains the MADS domain. Genomic sequence alignment revealed that *Sf-KjAG* and *Df-KjAG* share identical sequences except for an extra 2411 bp fragment inserted into intron 1 of *Df-KjAG* genomic DNA. Moreover, the inserted segment caused premature transcription termination and provided multiple alternative splice-acceptor sites; therefore, exons 2–6 of *Df-KjAG* mRNA are from the inserted segment. According to the unified classification system for eukaryotic transposable elements (Wicker et al., 2009), further identification revealed that the inserted fragment contains two target repeats (ATTTATAT) and two inverted repeats (TAGGGGTGGGC), suggesting a similarity to transposable elements. However, the inserted segment is neither a DNA transposon since it lacked sequences encoding transposase, nor a retro-transposon for the presence of introns, but the lack of long terminal repeats (**Figure 2B**). In addition, BLAST analysis of the inserted sequences revealed that a 108 bp sequence (from 1014 to 1122 bp of the inserted segment) has nearly 95% similarity with *P. mume* DNA-directed RNA polymerase V subunit 1 (LOC103330828). Another 118 bp fragment (from 1509 to 1627 bp of the inserted segment) was found to share nearly 93% similarity with *Malus × domestica* DNA-directed RNA polymerase V subunit 1-like (LOC103438702). However, the DNA-directed RNA polymerase encoded by the sequences is truncated, and an obvious frame-shift mutation was noted at the 1232 bp loci of the inserted segment.

### Southern Blotting and Distribution Detection of *Sf-KjAG* and *Df-KjAG* Transcripts

**FIGURE 3:**
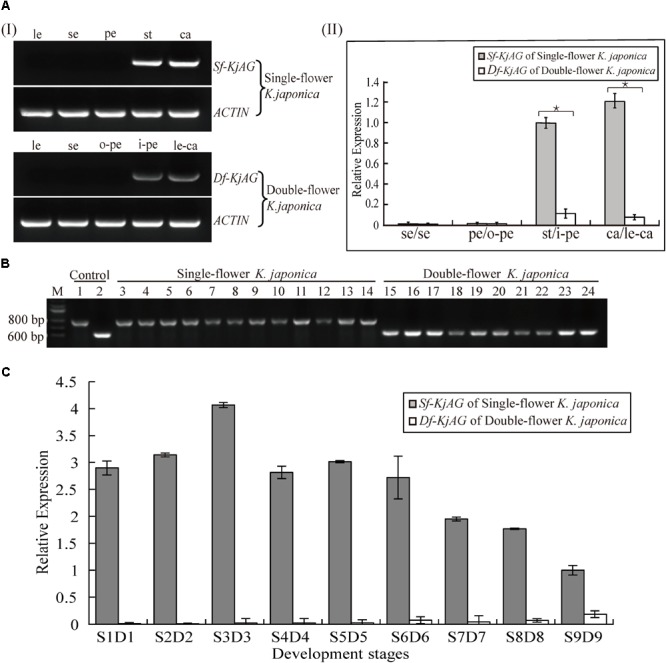
Expression pattern analysis of *Sf-KjAG* and *Df-KjAG*. **(Ai)** Tissue-specific expression pattern analysis of *Sf-KjAG* and *Df-KjAG* by semi-quantitative RT-PCR, **(Aii)** Expression pattern analysis of *Sf-KjAG* and *Df-KjAG* in different floral tissues by real-time quantitative PCR. le, leaves; se, sepals; pe, petals; st, stamens; ca, carpels; o-pe, outermost petals; i-pe, inner petals; le-ca, leaf-like carpels. **(B)** Distribution detection of *Sf-KjAG* and *Df-KjAG* in single- and double-flower *K. japonica* by multiplex PCR. Lane 1 is the PCR with plasmid which carries the *Sf-KjAG* full-length CDS as a template. Lane 2 is the PCR with plasmid which carries the *Df-KjAG* full-length CDS as a template. Lanes 3–14 are the PCR with the cDNA of single-flower *K. japonica* as templates. Lanes 15–24 are the PCR with the cDNA of double-flower *K. japonica* as templates. **(C)** Expression analysis of *Sf-KjAG* and *Df-KjAG* at different development stages by real-time quantitative PCR. Stars indicated *p* < 0.05 by student’s *t*-test.

The result of Southern blotting with an identical sequence of *Sf-KjAG* and *Df-KjAG* as a probe revealed that both *Sf-KjAG* and *Df-KjAG* are a single copy (**Figure 2C**). Multiplex PCR showed that the *Df-KjAG* transcript can be detected only in double-flower samples and *Sf-KjAG* transcript only in single-flower *K. japonica* samples (**Figure 3B**).

### Expression Patterns of *Sf-KjAG* and *Df-Kjag* in *K. japonica*

Semi-quantitative RT-PCR indicated that the *Sf-KjAG* transcript is strictly expressed in stamens and carpels and absent in leaves, sepals, and petals in single-flower *K. japonica* (**Figure 3A**). Similar to *Sf-KjAG, Df-KjAG* is expressed in the inner two whorls (inner petals and leaf-like carpels), but not in leaves, sepals, or the outermost petals. In addition, real-time quantitative PCR of different floral organ tissues showed that the expression level of *Df-KjAG* in whorls three and four was about 10 times lower than that in *Sf-KjAG* (**Figure 3A**).

Before real-time quantitative PCR on buds at different developmental stages, the stages of buds were determined using the traditional paraffin section method (Supplementary Figure S1). Real-time quantitative PCR analyses revealed that the expression level of *Sf-KjAG* increased gradually from the anther primordia stage (S1) to the primary sporogenous cell stage (S2) and reached a peak at the secondary sporogenous cell stage (S3). Further, its expression decreased and remained steady at the pollen mother cell stage (S4), late uninucleate stage (S5), and double nucleus stage (S6). Subsequently, the expression decreased gradually at the double nucleus stage (S7) and pollen maturation stage (S8) and remained lower until flowering (S9; **Figure 3C**). Compared with that of *Sf-KjAG*, the expression level of *Df-KjAG* was considerably lower in all development stages; in particular, during early flower development, the expression level of *Df-KjAG* was reduced by nearly four hundred times (**Figure 3C**).

### Functional Analysis of *Sf-KjAG* and *Df-KjAG* by Transforming *Arabidopsis*

In order to gain further insight into the function of *Sf-KjAG* and *Df-KjAG*, we constructed ectopic expression vectors with the genes under the control of the CaMV 35S promoter and transformed them into heterozygous *ag-1* mutant *Arabidopsis* plants by using *Agrobacterium*-mediated transformation. The transgenic plant genotypes were screened using the dCAPs method (Supplementary Figure S2).

**FIGURE 4:**
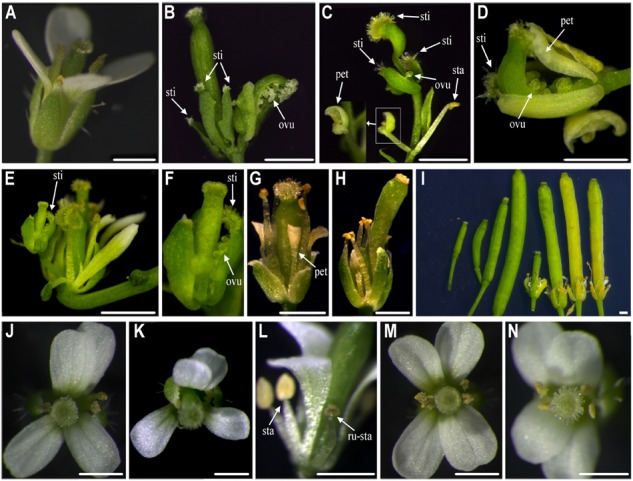
Functional analysis of *Sf-KjAG* and *Df-KjAG* in wild-type *Arabidopsis.* **(A)** The flower of the wild-type *Arabidopsis*. Comparison with wild-type *Arabidopsis*, transgenic *Arabidopsis* transformed with the pBI121 vector only (negative control) did not show any phenotype alteration (not shown) **(B–I)** Phenotype alterations of *35S::Sf-KjAG* transgenic *Arabidopsis* in the wild-type background. **(J–N)** Phenotype alterations of *35S::Df-KjAG* transgenic *Arabidopsis* in the wild-type background. **(B)** Carpelloid structures bear stigmatic papillae (sti) and ovules (ovu). **(C)** Carpelloid structures bear stigmatic papillae (sti) and ovules (ovu), and petal is converted into stamen-like structure. **(D)** Carpelloid structures bear stigmatic papillae (sti) and carpel is semi-fusion with ovules are exposed. **(E,F)** Sepals are transformed into carpel-like structures bearing stigmatic papillae (sti) and ovules are exposed. **(G)** The base of petals is converted to filament-like structures and sepals stretch outward. **(H)** The base of petals is converted to filament-like structures. **(I)** Four wild-type siliques are shown on the left and four *35S::Sf-KjAG* siliques are on the right; abscission of the outer three floral whorls (sepals, petals, and stamens) is inhibited. **(J,K)** The flowers of *35S::Df-KjAG* transgenic plants with 5 stamens. **(L)** Runtish stamen (ru-sta). **(M,N)** The flowers of *35S::Df-KjAG* transgenic plants with four stamens. sep, sepal; sti, stigmatic papillae; pet, petal; sta, stamen; ru-sta, runtish stamen; car, carpel; Bars = 1000 μm.

We obtained 56 *35S::Sf-KjAG* transgenic *Arabidopsis* in the wild-type background. Among these transgenic plants, 17 (30.36%) could be phenotypically distinguished from untransformed wild-type plants. From these 17 plants, 9 (16.07%) showed homeotic transformation from sepals into carpelloid structures bearing stigmatic papillae and ovules (**Figures 4B–F**), and petals into filament-like structures (**Figures 4C,G,H**). The remaining 8 (14.29%) plants showed flower phenotypes consisting of shortened petals converted to filament-like structures, and shrunken and revolute sepals (**Figures 4G,H**). In addition, the abscissions of the outer three floral whorls (sepals, petals, and stamens) were inhibited; thus, these flower tissues remained persistent until silique development (**Figure 4I**).

**FIGURE 5:**
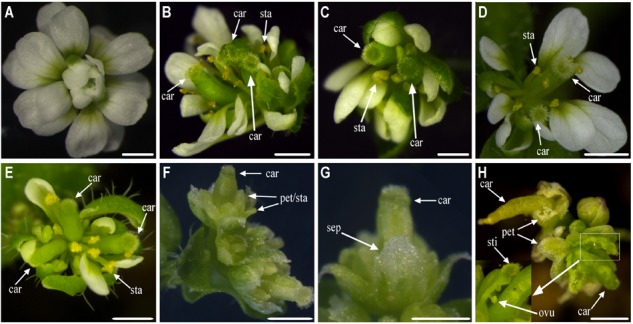
Functional analysis of *Sf-KjAG* in homozygous *ag-1* mutant *Arabidopsis*. **(A)** The flower of homozygous *ag-1* mutant *Arabidopsis*. Comparison with homozygous *ag-1* mutant *Arabidopsis*, transgenic *Arabidopsis* transformed with the pBI121 vector only (negative control) did not show any phenotype alteration (not shown). **(B–H)** The *35S::Sf-KjAG* transgenic *Arabidopsis* plants in the homozygous *ag-1* mutant background. **(B–E)** The flowers are recovered two or three carpels and multiple stamens. **(F,G)** The flowers are recovered one carpel, whose apexes of sepals are converted to white structures and stretching outward, and whose petals and stamens are indistinguishable. **(H)** The flowers are recovered one carpel and carpelloid structures bearing stigmatic papillae (sti) and ovules (ovu), and the petals decreased severely. sep, sepal; pet, petal; sta, stamen; car, carpel; sti, stigmatic papillae; ovu, ovule. Bars = 1000 μm.

We also obtained thirty-seven *35S::Sf-KjAG* plants in the homozygous *ag-1* mutant background. Compared with that in the homozygous *ag-1* mutant *Arabidopsis*, 13 (35.14%) plants exhibited obvious floral organ phenotype changes. In these lines, the missing stamens and carpels of the *Arabidopsis ag-1* mutants were rescued (**Figures 5B–H**). The number of recovered carpels varied from one to three per flower, and the number of recovered stamens ranged from zero to fourteen (**Figures 5B–H**). These stamens and carpels recovered in homozygous *ag-1* mutant prevented the formation of constantly nested sepals and petals in the flower center (**Figures 5B–H**). Moreover, the number of petals decreased (**Figures 5B–H**). Similarly, stigmatic papillae and ovules were also generated on sepals (**Figure 5H**). In addition, two (7.41%) plants exhibited splaying and white-edge sepals (**Figures 5F,G**).

Forty-three *35S::Df-KjAG* transgenic *Arabidopsis* plants in the wild-type background were obtained. None of these transgenic plants showed common phenotype alterations of *AG* homolog ectopic expression, such as the formation of carpelloid sepals and stamenoid petals. Instead, 10 (23.26%) plants showed phenotypic alterations involving stamen number reduction and runtish anthers (**Figure 4I**). Four (9.30%) plants maintained five stamens (**Figures 4J–L**), and other 6 (26.09%) plants had four stamens (**Figures 4M,N**). There were twenty-two *35S::Df-KjAG* transgenic plants in the homozygous *ag-1* mutant background that showed no phenotype rescue compared with those in the non-transgenic homozygous *ag-1* mutant.

**FIGURE 6:**
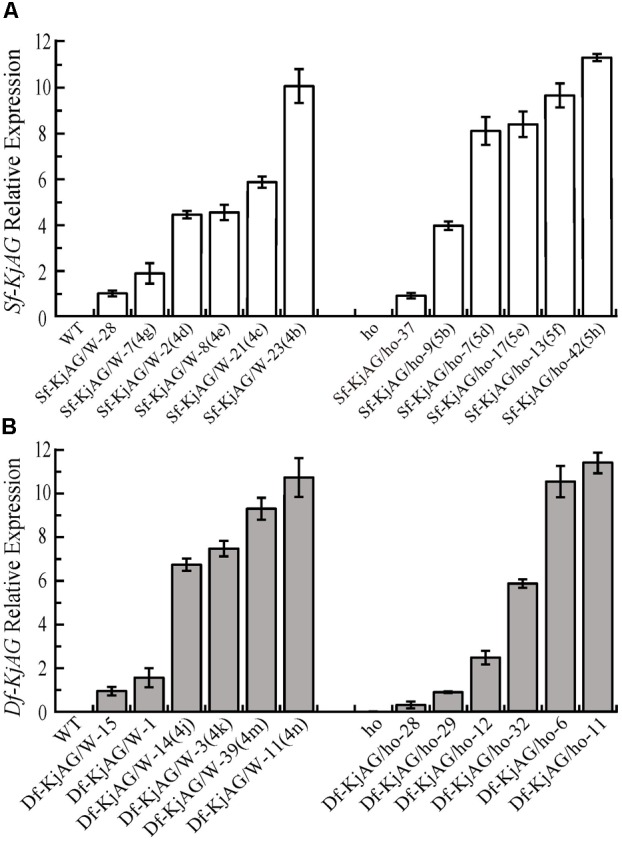
Quantitative analysis of *Sf-KjAG* and *Df-KjAG* expression in transgenic *Arabidopsis*. **(A)** The expression of *Sf-KjAG* in the wild-type and homozygous *ag-1* mutant transgenic *Arabidopsis*. **(B)** The expression of *Df-KjAG* in the wild-type and homozygous *ag-1* mutant transgenic *Arabidopsis*. WT are the transgenic plants with the pBI121 vector only (negative control) in the wild-type background; ho are the transgenic plants with the pBI121 vector only (negative control) in the homozygous *ag-1* mutant background. Sf-KjAG/W-28 and Sf-KjAG/ho-37 are the transgenic plants with no phenotype alteration in the wild-type and homozygous *ag-1* mutant background, respectively. Sf-KjAG/W-7(4g)/2(4d)/8(4e)/21(4c)/23(4b) and Sf-KjAG/ho-9(5b)/7(5d)/17(5e)/13(5f)/42(5h) are the *35S::Sf-KjAG* transgenic plants with obvious phenotype alterations in the wild-type and homozygous *ag-1* mutant background, respectively. Df-KjAG/W-15/1 are the *35S::Df-KjAG* transgenic plants with no phenotype alteration in the wild-type background. Df-KjAG/W-14(4j)/3(4k)/39(4m)/11(4n) are the *35S::Df-KjAG* transgenic plants with phenotypic alterations of stamen number reduction and runtish anthers in the wild-type background. Df-KjAG/ho-28/29/12/6/11 are the *35S::Sf-KjAG* transgenic plants in the homozygous *ag-1* mutant background. Numbers and letters in brackets are corresponding to those in **Figures 4, 5**.

Transgenic plants and relative expression levels were confirmed by quantitative RT-PCR. The results showed that *Sf-KjAG* expression levels were higher in transgenic plants with obvious transformation of sepals into carpelloid structures and petals into stamenoid petals than in those with no phenotypic alterations in the wild-type background (**Figure 6A**). In the homozygous *ag-1* mutant *Arabidopsis, Sf-KjAG* expression levels of were higher in the transformants with obvious phenotypic rescue of stamens and carpels than in those with no phenotypic alterations (**Figure 6A**). The *Df-KjAG* expression levels were higher in the transgenic plants with obvious phenotypic alterations of stamen number reduction and runtish anthers than in those with no phenotypic alterations in the wild-type background (**Figure 6B**). Further, although expression levels of *Df-KjAG* were also high in the homozygous *ag-1* mutant *Arabidopsis*, no phenotype alteration was noted in the transformants (**Figure 6B**).

### Interaction Pattern Comparison of Sf-KjAG and Df-KjAG

**FIGURE 7:**
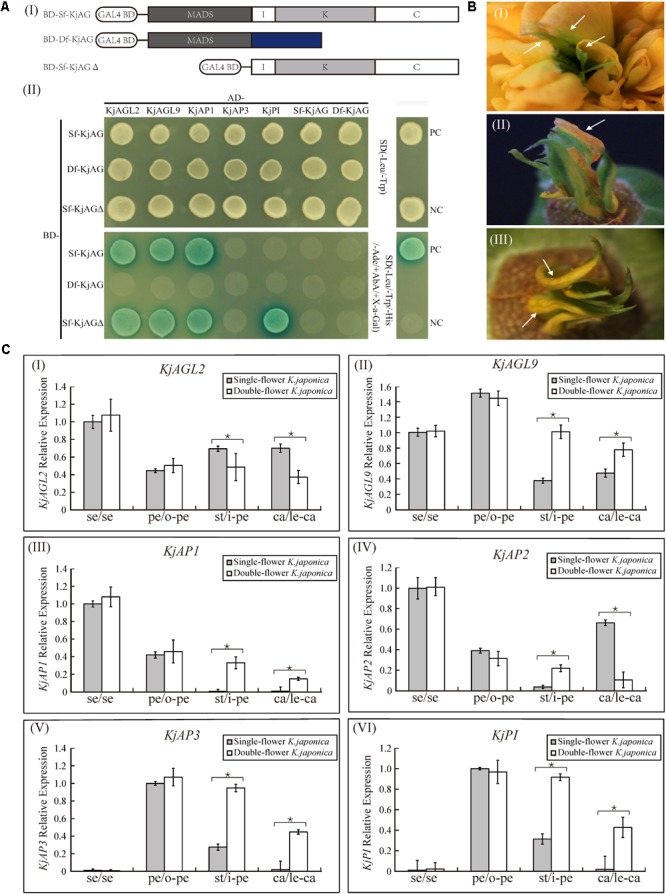
Interaction and expression analysis of floral organ identity genes in *K. japonica*. **(Ai)** The ligation schematic of Sf-KjAG, Df-KjAG and Sf-KjAGΔ (MADS domain of Sf-KjAG was deleted) to pGBKT7 vector with the GAL4-binding domain (BD). **(Aii)** Interaction analysis of Sf-KjAG, Df-KjAG, and Sf-KjAGΔ with KjAGL2, KjAGL9, KjAP1, KjAP3, KjPI, Sf-KjAG, and Df-KjAG by Y2H. PC, positive control; NC, negative control; Sf-KjAGΔ, MADS domain of Sf-KjAG is deleted; SD(-Leu/-Trp), synthetically defined medium lacking tryptophan and leucine; SD(-Leu/-Trp/-His/-Ade/+AbA/+X-a-Gal): synthetically defined medium lacking tryptophan, leucine, histidine and adenine and supplemented with Aureobasidin A and X-a-GAL. **(B)** Partial leaf-like carpels are adnate with petals in double-flower *K. japonica*. **(C)** Expression pattern analysis of *KjAGL2, KjAGL9, KjAP1, KjAP2, KjAP3*, and *KjPI* in single- and double-flower *K. japonica.* se, sepals; pe, petals; o-pe, outermost petals; st, stamens; i-pe, inner petals; ca, carpels; le-ca, leaf-like carpels. Stars indicated *p* < 0.05 by student’s *t*-test.

In previous studies, *AG* homologs in organ-specific programs required the formation of protein–protein complexes with the putative floral organ identity transcription factors (Coen and Meyerowitz, 1991; Theißen and Saedler, 2001; Liu et al., 2010). Our previous observation of the absence of I, K, and C domains in Df-KjAG protein led us to postulate that the mutant Df-KjAG might interrupt the interaction capability during floral development. In our Y2H experiment, we found that Sf-KjAG strongly interacted with KjAGL2, KjAGL9, and KjAP1, but not with KjAP3, KjPI, and Df-KjAG or homodimerized. None of these floral organ identity proteins could interact with Df-KjAG (**Figure 7A**). Interestingly, Sf-KjAGΔ (Sf-KjAG without the MADS domain) could interact with KjPI, which was not able to interact with Sf-KjAG (**Figure 7A**).

### Expression Pattern Comparison of A-, B-, and E-Class Genes in the Single- and Double-Flower *K. japonica*

To investigate whether the loss-of-function of *Sf-KjAG* could affect the expression patterns of other related putative floral organ identity genes, we performed real-time quantitative PCR to compare the expression patterns of *KjAGL2, KjAGL9, KjAP1, KjAP2, KjAP3*, and *KjPI* in both single- and double-flower *K. japonica.* In single-flower *K. japonica, KjAGL2* and *KjAGL9* were found to be expressed in all floral organs; *KjAP1* was specifically expressed highly in sepals and petals and little in stamens and carpels; *KjAP2* was expressed in all the whorls, whereas its level in stamen was relatively less; and *KjAP3* and *KjPI* expression levels were high in petals and stamens and little in sepals and carpels (**Figure 7C**). In double-flower *K. japonica*, the *KjAGL2* expression was decreased in inner petals and leaf-like carpels; the *KjAGL9* expression was increased in inner petals and leaf-like carpels; *KjAP1* was ectopically expressed in inner petals and leaf-like carpels; *KjAP2* expression was increased in the inner petals and decreased in leaf-like carpels; and both *KjAP3* and *KjPI* were expressed ectopically in leaf-like carpels (**Figure 7C**). Notably, the expression levels of these A-, B-, and E-class genes in inner petals seemed to be similar to those of normal petals in the single- and double-flower *K. japonica* (**Figure 7C**).

## Discussion

Double-flower varieties are more popular and attractive for higher ornamental values and more commercial interest. Although many double-flower phenotypes are noted in natural and domesticated species, the molecular mechanisms of double-flower formation in most varieties are not well understood. The gradually reduced petals centripetally and remnant filament-like and anther-like structures are the typical characteristics of extra petals transmuted from stamens in double-flower (Reynolds et al., 1984). Considering the morphology (**Figures 1G–J**), we speculated that the extra petals of double-flower *K. japonica* might be transmuted from stamens, and leaf-like structures are from carpels. Similarity of mutant phenotypes between *K. japonica* and other double-flower varieties caused by abnormal function or expression of *AG* homologous genes, such as *P. lannesiana* ‘speciosa’ and ‘Albo-rosea,’ *T. thalictroides ‘*Double White’ and *C. japonica*, the *K. japonica* double-flower formation might be related to the loss-of-function or aberrant expression of *AG* homologs.

A recent whole-genome duplication event might have occurred in Rosaceae 35.4–66.5 million years ago, and this was before pear (Maloideae, Rosaceae) and apple (Maloideae, Rosaceae) divergence, but not noted in strawberry (Rosoideae, Rosaceae; Wu et al., 2013). According to apple and strawberry genomes deposited in NCBI database, their *AG* orthologs are duplicated and single, respectively. Like *Sf-KjAG* and *Df-KjAG* (**Figure 2C**), both *AG* homologs in *P. lannesiana* ‘speciosa’ and ‘Albo-rosea’ (Rosaceae, Prunoideae) are a single copy (Liu et al., 2013). *K. japonica* and strawberry are grouped into Rosoideae, which might not have undergone the recent whole-genome duplication event that occurred before Maloideae divergence, but after Rosaceae divergence.

Sequence alignments revealed a transposon-like fragment insertion within intron one of *Df-KjAG* genomic DNA (**Figure 2**). However, partial structural elements related to transposition were lacking and two truncated coding sequences similar to polymerase genes were present in the transposon. Transposable elements often undergo rearrangement and truncations, such as abortive transposition events that lead to local rearrangements and deletions of internal sequences or nested insertions within other elements (Sanmiguel et al., 1996; Gorbunova and Levy, 1997; Yang et al., 2004; Krishnaswamy et al., 2010; Hirsch and Springer, 2017). Therefore, the transposition might be followed by transposable element rearrangements and truncations. Insertions of TEs within coding regions are generally mutagenic and result in strong loss-of-function, but insertions within introns or untranslated region are often tolerated and have minimal impact on transcription and splicing (West et al., 2014; Hirsch and Springer, 2017). In *Gentiana scabra*, an LTR-retrotransposon insertion within the intron 6 of *GsAG1* (C-class gene) did not generate novel transcripts (Nakatsuka et al., 2015). In ‘Rae Ime’ and ‘Spencer Seedless’ (apetalous and parthenocarpic apple variants), the splicing of *MdPI* (B-class gene) separately inserted by transposons within the fourth intron and the sixth intron was also not affected (Yao and Dong, 2001). However, in double-flower *K. japonica*, the transposon insertion within the intron of *AG* homolog caused premature transcription termination and generated novel transcript *Df-KjAG*, whose exons 2–6 were spliced from the inserted segment, which might be because the inserted transposon provided alternative splice-acceptor sites. In *T. thalictroides* cultivar ‘Double White,’ novel splicing also occurred at the alternative splice-acceptor sites provided by an transposon insertion within the exon 4 of *ThtAG1* (Galimba et al., 2012).

In *Arabidopsis*, the C-class gene *AG* plays key roles in determining the identity of stamens and carpels, limiting floral meristem determinacy and repressing A-class genes (Coen and Meyerowitz, 1991; Soltis et al., 2002; Heijmans et al., 2012). In the case of *AG* transgenic expression, homeotic transformation of sepals into carpels or carpelloid structures and petals into stamen-like structures in the wild-type *Arabidopsis*, and rescue of the stamens and carpels in the *ag* mutant *Arabidopsis* are typical phenotype alterations (Mizukami and Ma, 1992; Zhang et al., 2004; Lü et al., 2007). Such phenotype alterations have also been found in the *Sf-KjAG* transgenic *Arabidopsis*, which might suggest that *Sf-KjAG* plays the conserved function in specifying stamen and carpel identities, similar to those of *AG* in *Arabidopsis*. The I, K, and C domains are important to *AG* homologous genes, especially the C domain is essential for *AG* function (Egeacortines et al., 1999; Honma and Goto, 2001; Lamb and Irish, 2003; Tzeng et al., 2004). As expected, *Df-KjAG* did not show any putative *AG* homologous function; instead, its expression in the wild-type *Arabidopsis* reduced stamen number and affected anther development (**Figures 4J–N**), which indicated that the truncated *Df-KjAG* might inhibit normal *AG* function. Inhibition of normal *AG* function was also observed in transformants with truncated *AG* homologous genes transformed into *Arabidopsis* (Mizukami and Ma, 1992; Zhang et al., 2015). The *Df-KjAG* only contains the MADS domain, which is mainly responsible for target DNA binding (Shore and Sharrocks, 1995; Riechmann and Meyerowitz, 1997; Yang and Jack, 2004; Liu et al., 2010). Therefore, we speculated that the reduced stamen number and runtish stamens might be caused by competitive inhibition against the endogenous *AG* of *Arabidopsis* at downstream MADS domain target DNA in *Df-KjAG* transformants. These results suggested that *Df-KjAG* has lost the *AG* homologous function of reproductive organ determinacy. Loss-of-function or aberrant expression of *AG* orthologous genes often promotes double-flower formation, such as in *Arabidopsis, C. japonica, T. thalictroides*, Japanese morning glory, *N. damascena*, and tomato (Bowman, 1997; Benedito et al., 2004; Dong et al., 2007; Liu et al., 2013; Tanaka et al., 2013; Noor et al., 2014; Sun et al., 2014; Nakatsuka et al., 2015; Wang et al., 2015). Therefore, we speculated that the loss-of-function of *Df-KjAG* induces double-flower formation in *K. japonica*.

In single-flower *K. japonica, Sf-KjAG* was only expressed in stamens and carpels, which was consistent with the tissue expression patterns of *AG* and other *AG* homologous genes in species such as *T. rupestris* and *P. lannesiana* (Lü et al., 2007; Liu et al., 2013). In double-flower *K. japonica*, although *Df-KjAG* has lost the C-class function, its expression was still limited to inner whorls, which might be attributed to the repressors of *AG* homologs. In *Arabidopsis*, the inner whorl-specific activation of *AG* was achieved by the repressors of *AG*, such as *BELLRINGER* (*BLR*), *LEUNIG* (*LUG*), and *SEUSS* (*SEU*). *BLR* and *SEU*/*LUG* complex can be recruited to *AG cis*-regulatory region (such as the second intron of *AG*) and prevents the ectopic *AG* expression in the two outer whorls of the flower (Franks et al., 2002; Bao et al., 2004; Sridhar et al., 2006). However, the *Df-KjAG* expression level was significantly lower than that of *Sf-KjAG* in the inner two whorls (**Figure 3A**) and at all development stages (**Figure 3C**). Similar expression patterns were also found in *T. thalictroides ‘*Double White,’ *Cyclamen persicum*, and double-flower *Arabidopsis* (de Folter et al., 2005; Gómezmena et al., 2005; Tanaka et al., 2013). In *Arabidopsis*, AG can form protein heterodimers with SEP3 to act in a positive auto-regulatory loop that maintains and amplifies *AG* expression (Honma and Goto, 2001; de Folter et al., 2005; Gómezmena et al., 2005; Sridhar et al., 2006; Alvarez-Buylla et al., 2010). In our Y2H analyses, Df-KjAG protein could not form protein complexes with KjAGL9, an SEP3 orthologous protein (**Figure 7A**). Therefore, the reduced *Df-KjAG* levels might be attributed to the fact that Df-KjAG protein cannot form a protein complex to maintain and amplify *Df-KjAG* expression. Loss-of-function of *Df-KjAG* also changed the expression patterns of *KjAGL2* and *KjAGL9* (putative E-class genes), *KjAP1* and *KjAP2* (putative A-class genes), and *KjAP3* and *KjPI* (putative B-class genes) in whorls three (inner petals) and four (leaf-like carpels) of double-flower *K. japonica*. Although E-class genes may be the upstream genes of *AG* homologs, expression pattern modifications of E-class genes (*AGL2* in *Arabidopsis*, and *DEFH200* and *DEFH72* in *Antirrhinum majus*) have been reported previously in the *Arabidopsis ag* mutant and *Antirrhinum majus plena* (C-class gene) mutant (Flanagan and Ma, 1994; Davies et al., 1996). These findings might suggest that the expression regulation among floral organ identity genes is not strictly hierarchical, and the expression of upstream genes can also be affected by the latter genes. The expression pattern changes of A-, B-, and E-class genes are broadly consistent with the mutant phenotypes observed in double-flower *K. japonica*. According to the ABCE model, A- and E-class genes are responsible for sepal development; A-, B-, and E-class, for petal development; B-, C-, and E-class for stamen development; and C- and E-class for carpel development (Coen and Meyerowitz, 1991; Soltis et al., 2002; Liu et al., 2010; Heijmans et al., 2012; Soltis and Soltis, 2014). In double-flower *K. japonica*, transformation of stamens into petals (**Figure 1**) might lead to the similar expression of A-, B-, and E-class genes between inner petals and normal petals (**Figure 7C**); ectopic expression of B-class genes as well as expression of A- and E-class genes might be responsible for the adnation of petals with leaf-like carpels in whorl four (**Figure 7C**). In double-flower Japanese gentian, expression patterns of its A-class genes (*GsAP1* and *GsAP2*) were also modified in whorls 3 and 4 (Nakatsuka et al., 2015). In double-flower cultivar ‘Jinpanlizhi,’ expression analysis was also shown that B-class genes are expressed in whorl 4 (Sun et al., 2014). These findings might suggest that the expression patterns of floral organ identity genes in different whorls are maintained by mutual regulation, and aberrant expression of certain genes might lead to boundary shift or expression modification of other floral organ identity genes.

The correct function of *AG* homologs in organ-specific programs requires the formation of protein–protein complexes with the putative floral organ identity transcription factors (Coen and Meyerowitz, 1991; Theißen and Saedler, 2001; Liu et al., 2010). The protein–protein interaction patterns of Sf-KjAG are consistent with those of *AG* in *Arabidopsis* and *AG* orthologous genes in other species such as *Petunia hybrida* and *Antirrhinum majus* (Davies et al., 1996; Favaro et al., 2003; Ferrario et al., 2003; Leseberg et al., 2008; Liu et al., 2010), which might suggest that *Sf-KjAG* plays the analogous function in floral organ development, similar to that of other *AG* homologous genes. The I domain is shown to be responsible for the selective formation of DNA-binding dimers; the K domain, for protein–protein interactions; and C domain, for the formation of higher-order complexes and functional specificity (Shore and Sharrocks, 1995; Riechmann et al., 1996; Riechmann and Meyerowitz, 1997; Honma and Goto, 2001; Yang and Jack, 2004; Kaufmann et al., 2005; Liu et al., 2010). Thus, the lack of I, K, and C domains of Df-KjAG may well explain the loss of normal interaction capability and function in specifying stamen and carpel determinacy. In addition, Sf-KjAGΔ (MADS domain of Sf-KjAG was deleted) can still interact with KjAP1, KjAGL2, and KjAGL9, which might indicate that the MADS domain of Sf-KjAG seems to be dispensable in the dimerization with KjAP1, KjAGL2, and KjAGL9, and their dimerization mainly depends on I, K, and C domains (**Figure 7A**). Once the MADS domain of Sf-KjAG protein was deleted, the remaining domains could generate strong interactions with KjPI, which cannot interact with Sf-KjAG (**Figure 7A**). This might suggest that the MADS domain of Sf-KjAG also plays a role in specific- or selective-dimerization, besides the main function in binding to target DNA.

## Conclusion

Although we did not verify the double-flower formation directly in *K. japonica*, considering that woody plants have lengthy generation periods and are not amenable to the standard techniques of functional genomics, multiple evidences have been provided to show that the double-flower formation of *K. japonica* is likely to result from the loss-of-function of *AG* homologous gene by a transposon insertion into an intron of the *AG* homologous genomic DNA. The transposon insertion caused premature transcription termination and generated normal transcript by aberrant splicing at alternative splice-acceptor sites, and subsequently resulted in prominent loss in protein–protein interaction and function. Further, the loss-of-function of *Df-KjAG* would affect the expression patterns of itself and other related floral organ identity genes. Furthermore, we showed that a transposon within introns is caused by flower homeotic mutation, which provides further evidences supporting the potential roles of these elements in plant domestication and evolution.

## Author Contributions

FC conceived and directed this study. JM and XS performed the experiments, analyzed the data, and wrote the manuscript. ZL, DZ, WL, HL, YW, and ZH provided suggestions and revised the manuscript.

## Conflict of Interest Statement

The authors declare that the research was conducted in the absence of any commercial or financial relationships that could be construed as a potential conflict of interest.
