# Supplementary material for: Isolation and Characterization of AGAMOUS-Like Genes Associated With Double-Flower Morphogenesis in Kerria japonica (Rosaceae)
Source: Front Plant Sci. 2018 Jul 12;9:959. doi: 10.3389/fpls.2018.00959 (PMC6052346; doi:10.3389/fpls.2018.00959)
Supplement: Supplementary file 1 [file Table_1.DOC]

**Table S1.** A list of all primers used for gene cloning, semi RT-PCR, qRT-PCR and genotype identification in this study.

| Primer | Primer sequences(5’ to 3’) |
| --- | --- |
| 3’AGGSPF1 | AATGGGTTGCTCAAGAAGGCCTATG |
| 5'AGGSPR1 | TCTTGGCGTGGATTGTACTGATGA |
| Sf/Df-KjAG-F | TCTAGACATCTATGGCTTATGAAAACAAATC |
| Sf-KjAG-R | CCCGGGAGAAACGACGGTCCAAGCAGATA |
| Df-KjAG-R | GAGCTCAATATGTACACTAGATGTCAATC |
| KjAG-GeDNA-F | ATGGCTTATGAAAACAAATCCATGTC |
| KjAG-GeDNA-R | ATACTTGACAACGTGCTTGTAGCTC |
| Kj-sou-F | TTCTATCAAGCACCTCTCTTGGCC |
| Kj-sou-R | AGAAACGACGGTCCAAGCAGA |
| RT-Sf/Df-KjAG-F | AACCAAAAGCTTGCATCTATGGC |
| RT-Sf-KjAG-R | CAGATAATTATTGCGGGAACCCA |
| RT-Df-KjAG-R | GCATCCGCTTCAATTCGCTAACA |
| RT-Kjactin-F | TGTGAGTCACACTGTGCCAA |
| RT-Kjactin-R | GCAGCTTCCATTCCAATGAG |
| qSf/Df-KjAG-F | AGAGGAGCCAGCAGAACATAAACG |
| qSf-KjAG-R | AGAAACGACGGTCCAAGCAGATAAT |
| qDf-KjAG-F | CTGTTGAAGTGGAAGCGAAAGCGAG |
| qDf-KjAG-R | ACACTAGATGTCAATCCCTCCAAGA |
| qKjactin-F | TTGACCTTGCTGGTCGTGACCTC |
| qKjactin-R | CTGGTAACTCATAGCTCTTCTC |
| Aractin-F | CGTATGAGCAAGGAGTACAC |
| Aractin-R | CACATCTGTTGGAAGGTGCT |
| ag-f | GATATATTAACATATGTTGATAAATCACTTA |
| ag-r | AATCAACTTCCTGCTTAATCGGT |
| Aractin-F | GCTCTTCCACATGCTATTCTG |
| Aractin-R | TTCCATCTCTTGCTCGTAGTCA |
| qKjPI-F | CAAGTATCCGGGACAAGCAGTC |
| qKjPI-R | CGCGAAAGGAAAAAGGTATCTG |
| qKjAP3-F | CTCAGACGGAGACCTGCAAGA |
| qKjAP3-R | GTGGAGGCGAAAAGCAAACAAA |
| qKjAP1-F | AGATCAAGGAGAACGAGAAGGC |
| qKjAP1-R | CGAGGTGGCATGAATAGAGAGG |
| qKjAP2-F | TGATCTCTCACTGTCAACGAACG |
| qKjAP2-R | GAGAAATGGTCATGATGGTCAAG |
| qKjAGL2-F | GAAGCAAACAGGGATTTGACAATC |
| qKjAGL2-R | AGACCCCACAGTAGAGTAGCC |
| qKjAGL9-F | CAACTGAATCCAAATGCTGACGAG |
| qKjAGL9-R | CATCGCACGTACACCTTTTATTATC |
| AD-KjAP3-F | GAATTCATGGGTCGTGGGAAGATTGAGA |
| AD-KjAP3-R | CCCGGGACCGAGTCCAAGTCTAAGTCC |
| AD-KjAP1-F | CCCGGGCATGGGGAGGGGTAGGGTTCAGC |
| AD-KjAP1-R | GGATCCGCGACCACATACCTTAGCAAT |
| AD-KjPI-F | GAATTCATGGGGAGGGGCAAGATTGAGA |
| AD-KjPI-R | GGATCCGTTGATAGCACTTCCTGACAG |
| AD-KjAGL2-F | GAATTCATGGGGAGGGGAAGAGTGGAAC |
| AD-KjAGL2-R | GGATCCTACTGCCAGCAAAAGAAGCAT |
| AD-KjAGL9-F | GAATTCATGGGGAGGGGGAGGGTGGAATTG |
| AD-KjAGL9-R | GAGCTCTCAAGGCAACCATCCCGGCATGTA |
| AD/BD-Sf/Df-KjAG-F | GAATTCATGGCTTATGAAAACAAATCC |
| AD/BD-Sf-KjAG-R | GGATCCAACGACGGTCCAAGCAGATAA |
| AD/BD-Df-KjAG-R | GGATCCAAGAGACTTCCTCCATTTCCT |
| AD/BD-Sf-KjAGΔ-F | GAATTCATGGCCAACAACAGTGTCAGAGC |
