# Supplementary material for: Isolation and Characterization of AGAMOUS-Like Genes Associated With Double-Flower Morphogenesis in Kerria japonica (Rosaceae)
Source: Front Plant Sci. 2018 Jul 12;9:959. doi: 10.3389/fpls.2018.00959 (PMC6052346; doi:10.3389/fpls.2018.00959)
Supplement: Supplementary file 2 [file Table_2.DOC]

**Table S2.** All the MADS-box proteins in protein sequences referred in this paper.

| Protein | Species | Accession number |
| --- | --- | --- |
| AG | *Arabidopsis thaliana (thale cress)* | P17839 |
| AGL1/SHP1 | *Arabidopsis thaliana (thale cress)* | NP_191437 |
| AGL5/SHP2 | *Arabidopsis thaliana (thale cress)* | NP_850377 |
| BepeMADS | *Betula pendula* | CAB95649 |
| CmMADS2 | *Castanea mollissima* | AAZ77747 |
| CoavMADS1 | *Corylus avellana* | AAD03486 |
| CoheMADS1 | *Corylus heterophylla* | AEU08497 |
| CUM1 | *Cucumis sativus* | AAC08528 |
| FBP6 | *Petunia x hybrida* | CAA48635 |
| GhMADS3 | *Gossypium hirsutum* | AAL92522 |
| HmAG | *Hydrangea macrophylla* | BAG74745 |
| JacuAG | *Jatropha curcas* | AEA11211 |
| MadoAG_x1 | *Malus domestica* | XP_008371525 |
| MadoMADS | *Malus domestica* | CAC80858 |
| MASAKO C1 | *Rosa rugosa* | BAA90744 |
| MASAKO D1 | *Rosa rugosa* | BAA90743 |
| PLE | *Antirrhinum majus* | AAB25101 |
| pMADS3 | *Petunia x hybrida* | CAA51417 |
| PperSHP | *Prunus persica* | ABG75908 |
| PpMADS4 | *Prunus persica* | AAU29513 |
| PravAG | *Prunus avium* | BAP90526 |
| PrpeAG | *Prunus persica* | XP_007211925 |
| PrpmAG_x1 | *Prunus mume* | XP_008225406 |
| PrpsAG | *Prunus pseudocerasus* | AIU94283 |
| PrseAG | *Prunus serrulata var. lannesiana* | AEA48983 |
| PrseSHP | *Prunus serrulata var. lannesiana* | ADG45819 |
| PsAG | *Prunus serotina* | ACH72974 |
| PybrAG_x1 | *Pyrus x bretschneideri* | XP_009351902 |
| STAG1 | *Fragaria x ananassa* | AAD45814 |
| TAG1 | *Solanum lycopersicum* | AAA34197 |
| ThcaMADS1 | *Theobroma cacao* | XP_007025250 |
| ThcaMADS2 | *Theobroma cacao* | XP_007025251 |
| TrAG | *Taihangia rupestris* | ABB59994 |
| TrSHP | *Taihangia rupestris* | ABB59995 |
| AP3 | *Arabidopsis thaliana* | NP_191002 |
| PI | *Arabidopsis thaliana* | NP_197524 |
| AGL2/SEP1 | *Arabidopsis thaliana* | NP_568322 |
| AGL4/SEP2 | *Arabidopsis thaliana* | NP_568322 |
| AGL9/SEP3 | *Arabidopsis thaliana* | NP_850953 |
| AGL3/SEP4 | *Arabidopsis thaliana* | NP_849930 |
| AGL79 | *Arabidopsis thaliana* | NP_189645 |
| AGL8/euFUL | *Arabidopsis thaliana* | NP_568929 |
| AP1 | *Arabidopsis thaliana* | NP_177074 |
| STK | *Arabidopsis thaliana* | NP_192734 |
| FBP11 | *Petunia hybrid* | CAA57445 |
| FBP7 | *Petunia hybrid* | CAA57311 |
| NdAG1 | *Nigella damascena* | ALM95507 |
| NdAG2 | *Nigella damascena* | ALM95508 |
| CyAG | *cycas edentata* | AAM74074 |
| pMADS3 | *Petunia hybrid* | Q40885 |
| CjAG | *Camellia japonica* | AIP87050 |
| MastAG | *Magnolia stellata* | JQ326243 |
| ThtAG1 | *Thalictrum thalictroides* | ALF08434 |
| DEFH200 | *Antirrhinum majus* | CAA64743 |
| DEFH72 | *Antirrhinum majus* | CAA64742 |
| LIPLESS1 | *Antirrhinum majus* | AAO52746 |
| LIPLESS2 | *Antirrhinum majus* | AAO52747 |
| GmAP1 | *Glycine max* | XP_014627678 |
| PaAP1 | *Prunus avium* | XP_021806356 |
| PpMADS1 | *Prunus persica* | ABU63953 |
| PaAP2L1 | *Picea abies* | AAG32658 |
| PaAP2L2 | *Picea abies* | AAG32659 |
| PaAP2L3 | *Picea abies* | DQ089703 |
| KjAGL2 | *Kerria japonica* | MH161198 |
| KjAGL9 | *Kerria japonica* | MH161199 |
| KjAP1 | *Kerria japonica* | MH161200 |
| KjAP2 | *Kerria japonica* | MH161201 |
| KjAP3 | *Kerria japonica* | MH161202 |
| KjPI | *Kerria japonica* | MH161203 |
| Sf-KjAG | *Kerria japonica* | KC476500 |
| Sf-KjAG genomic DNA | *Kerria japonica* | KT884659 |
| Df-KjAG | *Kerria japonica* | KT884658 |
| Df-KjAG genomic DNA | *Kerria japonica* | KT884660 |
