# Supplementary material for: Isolation and Characterization of AGAMOUS-Like Genes Associated With Double-Flower Morphogenesis in Kerria japonica (Rosaceae)
Source: Front Plant Sci. 2018 Jul 12;9:959. doi: 10.3389/fpls.2018.00959 (PMC6052346; doi:10.3389/fpls.2018.00959)
Supplement: Supplementary file 3 [file Image_1.PDF]

Figure S1. Flower development stage confirmation by paraffin section.

(A)

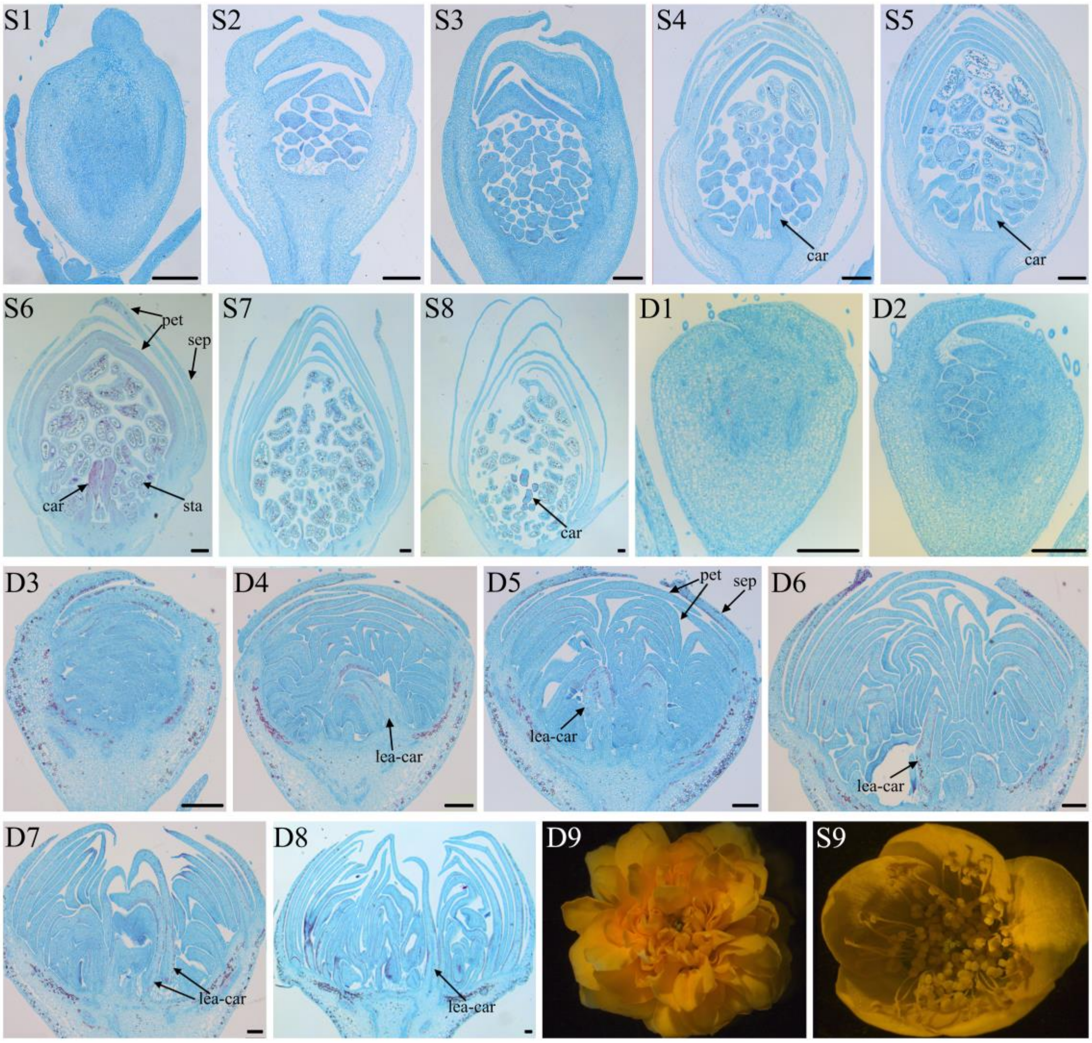

(B)

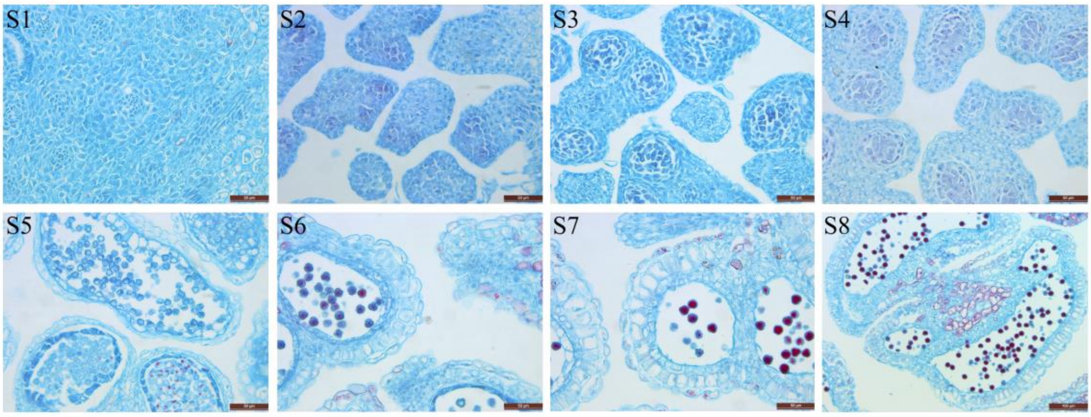

(A) The whole graphs of floral buds at different developmental stages.

(B) The partial enlarged view of floral buds at different developmental stages.

S1-S9: Flower buds at different developmental stages in single-flower *K. japonica*; D1-D9:

Flower buds at different developmental stages of double-flower *K. japonica*; S1: Single-flower at the anther primordia developmental stage; S2: Single-flower at the primary sporogenous cell stage; S3: Single-flower at the stage of secondary sporogenous cells; S4: Single-flower at the stage of pollen mother cells; S5: Microspores at the late uninucleate stage; S6: Single-flower at the double nucleus stage, and petals at the rapid elongation stage; S7: Single-flower at the double nucleus stage and petals at continuous elongation; S8: Single-flower at the pollen mature stage; S9: Opened single-flower. D1-D9: The developmental stages of double flower corresponding to S1-S9. carpel, ca; leaf-like carpel, le-car; Bars = 200μm.
