# Supplementary material for: Isolation and Characterization of AGAMOUS-Like Genes Associated With Double-Flower Morphogenesis in Kerria japonica (Rosaceae)
Source: Front Plant Sci. 2018 Jul 12;9:959. doi: 10.3389/fpls.2018.00959 (PMC6052346; doi:10.3389/fpls.2018.00959)
Supplement: Supplementary file 4 [file Image_2.PDF]

Figure S2. Genotyping of wild-type, heterozygote and homozygous *ag-1* mutant transgenic *Arabidopsis* by dCAPS.

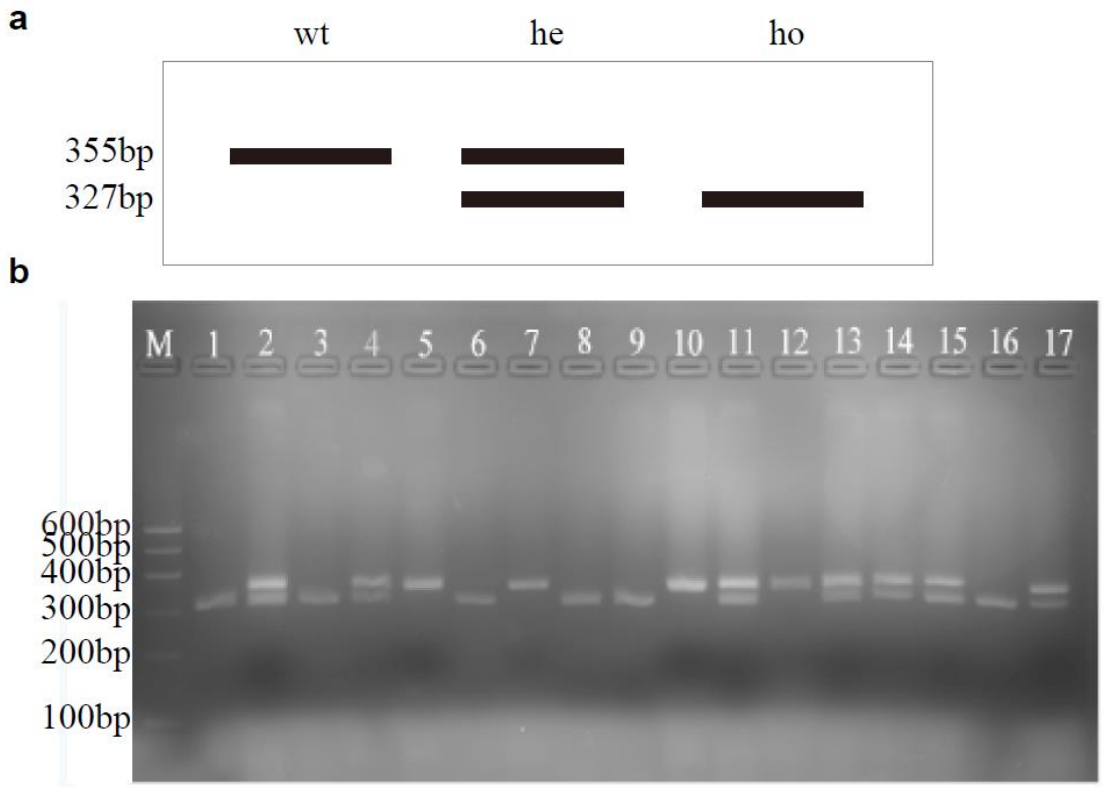

a: The diagram of genotype identification of *ag-1* mutant *Arabidopsis*. The amplicon of homozygous *ag-1* mutant *Arabidopsis* plants was cleaved by *Afl* II to reduce it to a 324-bp fragment; The amplification product of heterozygote *AG/ag-1* mutant *Arabidopsis* plants was cleaved by *Afl* II to produce two fragments (324-bp and 354-bp in length); The wild-type amplicon was cleaved to get a 354-bp fragment by *Afl* II.

b: The genotype identification results of a portion of transgenic plants.
