# Supplementary material for: Isolation and Characterization of AGAMOUS-Like Genes Associated With Double-Flower Morphogenesis in Kerria japonica (Rosaceae)
Source: Front Plant Sci. 2018 Jul 12;9:959. doi: 10.3389/fpls.2018.00959 (PMC6052346; doi:10.3389/fpls.2018.00959)

Figure S3. Phylogenic analyses of MADS-box genes from *K. japonica* and represented MADS-box genes from other eudicots.

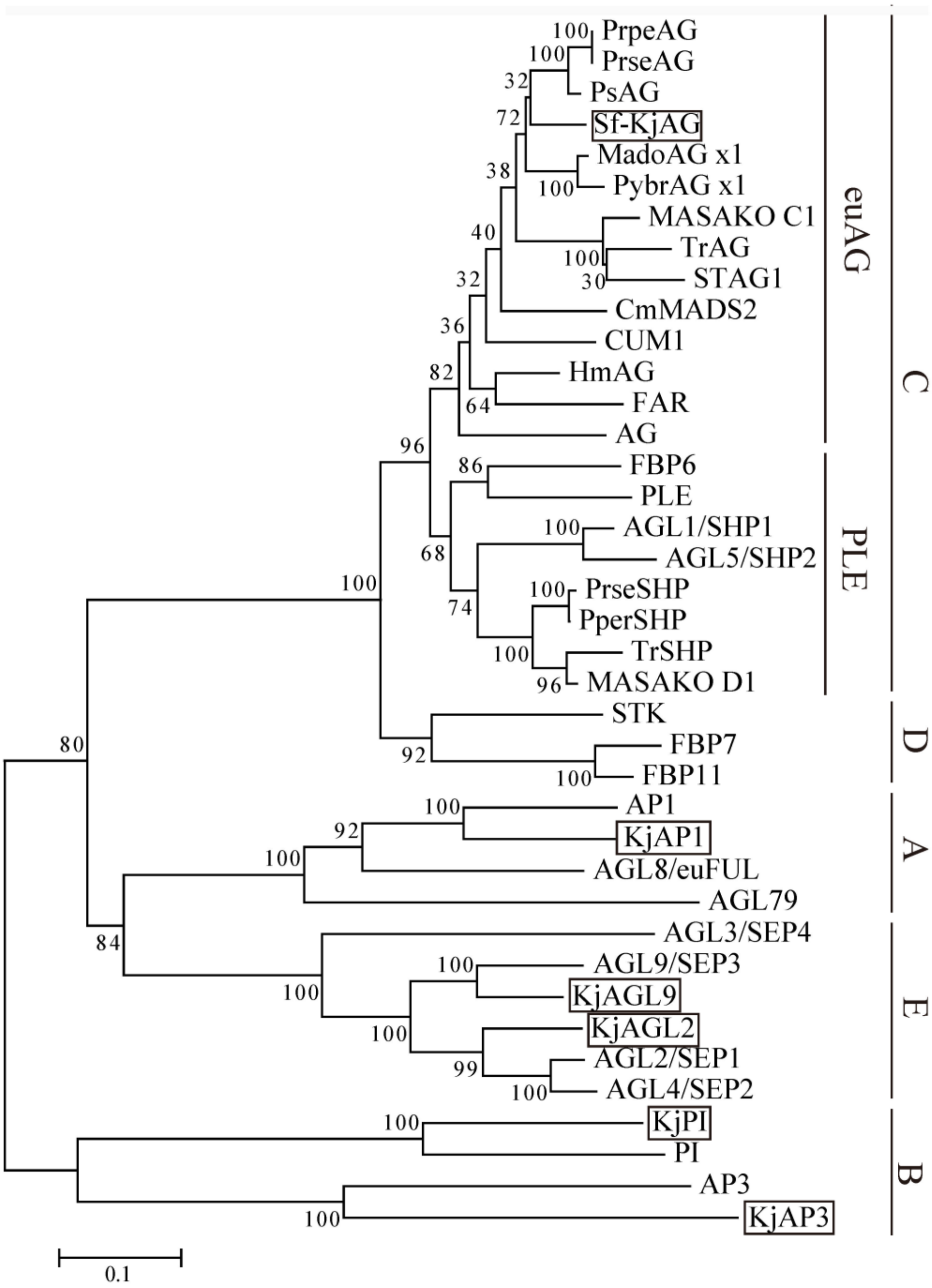

Supplement: Supplementary file 5 [file Image_3.PDF]
