# Supplementary material for: Isolation and Characterization of AGAMOUS-Like Genes Associated With Double-Flower Morphogenesis in Kerria japonica (Rosaceae)
Source: Front Plant Sci. 2018 Jul 12;9:959. doi: 10.3389/fpls.2018.00959 (PMC6052346; doi:10.3389/fpls.2018.00959)
Supplement: Supplementary file 6 [file Image_4.PDF]

Figure S4. Sequence alignments of Sf-KjAG protein and the other AG-related MADS-box proteins.

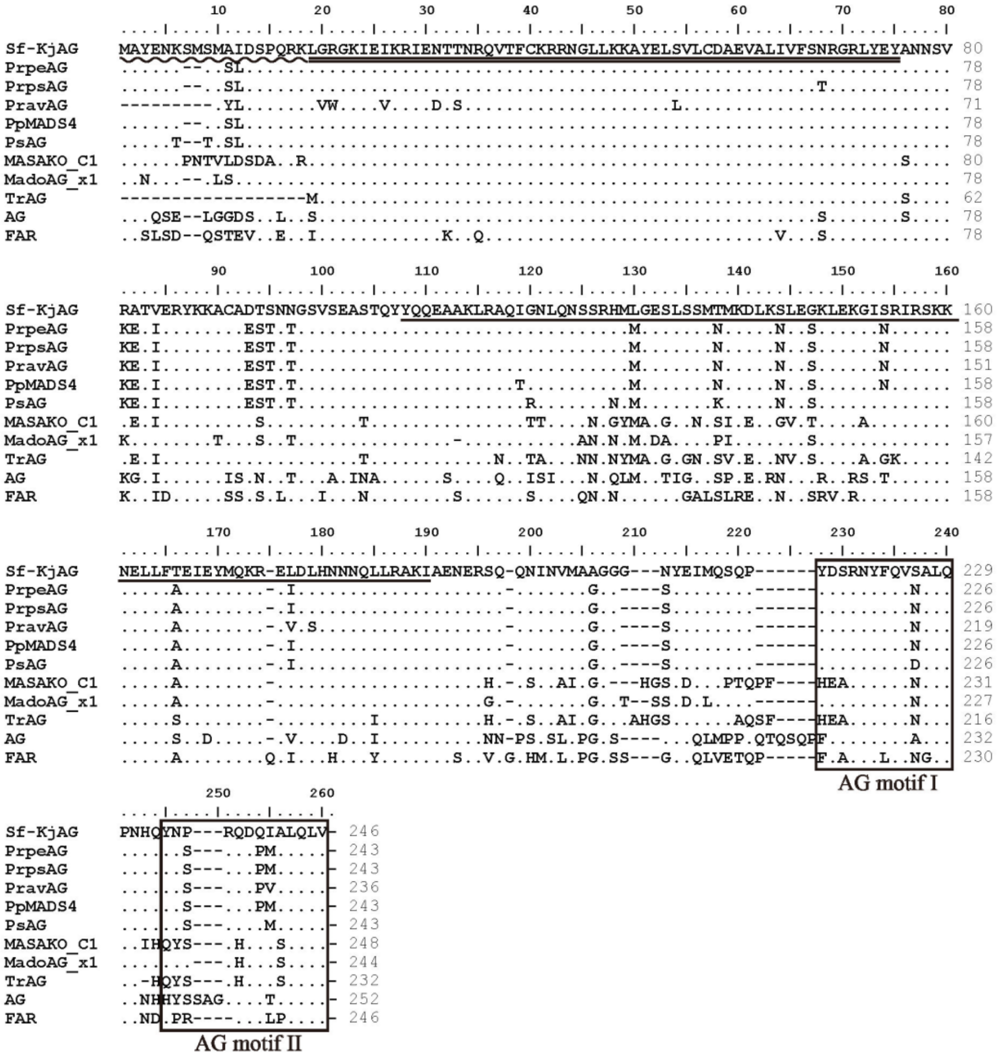

The wave line region represents the N-terminal extension. The double underline region represents the MADS domain. The region between double underline and single underline represents the I domain. The single underline region represents the K domain. The region after single underlined region represents the C domain. The AG motifs I and II are boxed. Amino acid residues identical to Sf-KjAG are indicated as dots. To improve the alignment, dashes were introduced into the sequence.
